# Non-invasive Stereotactic Body Radiation Therapy for Refractory Ventricular Arrhythmias: Venturing into the Unknown

**DOI:** 10.19102/icrm.2022.130202

**Published:** 2022-02-15

**Authors:** Justin Hayase, Robert Chin, Minsong Cao, Peng Hu, Kalyanam Shivkumar, Jason S. Bradfield

**Affiliations:** ^1^UCLA Cardiac Arrhythmia Center, Ronald Reagan UCLA Medical Center, Los Angeles, CA, USA; ^2^Radiation Oncology, Ronald Reagan UCLA Medical Center, Los Angeles, CA, USA; ^3^Department of Radiological Services, Ronald Reagan UCLA Medical Center, Los Angeles, CA, USA

**Keywords:** Catheter ablation, stereotactic body radiation therapy, ventricular arrhythmias

## Abstract

Stereotactic body radiation therapy (SBRT) is a promising new method for non-invasive management of life-threatening ventricular arrhythmias. Numerous case reports and case series have provided encouraging short-term results suggesting good efficacy and safety, but randomized data and long-term outcomes are not yet available. The primary hypothesis as to the mechanism of action for SBRT relates to the development of cardiac fibrosis in arrhythmogenic myocardial substrate; however, limited animal model data offer conflicting insights into this theory. The use of SBRT for patients with refractory ventricular arrhythmias is rapidly increasing, but ongoing translational science work and randomized clinical trials will be critical to address many outstanding questions regarding this novel therapy.

## Introduction

Modern techniques and technology for the catheter ablation of ventricular tachycardia (VT) continue to improve and remain a cornerstone of therapy for the management of these life-threatening arrhythmias.^1^ However, radiofrequency ablation is a complex and often protracted procedure. Standard ablation may employ both an endocardial and an epicardial access approach and require mapping during hemodynamically unstable arrhythmias, and the case may take several hours to achieve a successful outcome. Many patients may also require multiple procedures. Additionally, patients with these arrhythmias often suffer from multiple comorbid conditions, further increasing the peri-procedural risk. In recent years, stereotactic body radiation therapy (SBRT) as a non-invasive treatment option for recalcitrant ventricular arrhythmias has garnered much attention. Multiple case reports and case series have been published, with promising, although variable, degrees of success.^2–7^

## Mechanism of action

Ionizing radiation has long been used in cancer therapeutics, and, for many years, the focus on SBRT delivery has been to minimize cardiac exposure to radiation. As such, insights as to the potential antiarrhythmic benefits of SBRT are not yet fully understood. Scar-mediated VT depends on surviving myocyte bundles within the regions of fibrosis, which serve as the substrate for unidirectional conduction block to promote reentrant arrhythmia mechanisms.^8^ Catheter ablation targets these critical isthmuses, and a comprehensive scar homogenization approach that aims to eliminate all potential VT circuits may result in improved outcomes based on the available data.^9^ Whether and how SBRT exerts its anti-arrhythmic mechanism of action similar to catheter ablation is not yet known.

Early autopsy data have demonstrated the long-term effects of excessive radiation inadvertently delivered to the heart during cancer treatments.^10^ The primary histopathological observation in these cases was the widespread myocardial fibrosis. It stands to reason that a primary hypothesis for the SBRT mechanism of action in the elimination of VT is therefore a scar-homogenization effect and elimination of VT isthmuses, similar to that achieved with catheter ablation. A number of animal model studies have supported this hypothesis.^11,12^ A recent human study examined 4 previously irradiated explanted hearts following cardiac transplantation.^13^ Subendocardial necrosis with surrounding fibrosis was seen on histopathological analysis, lending further support to this theory.

## Patient selection

Since the first case report of a patient at prohibitive risk for catheter ablation due to numerous medical comorbidities,^14^ interest in SBRT as a treatment for refractory ventricular arrhythmias has grown. The capability of SBRT to be delivered in an ambulatory, non-sedated patient in a single fraction during a session of 15–30 minutes is an incredibly attractive option. As such, there are few circumstances that might prevent a patient from feasibly undergoing SBRT. Exclusions include pregnancy, hemodynamic instability, or excessive risk to nearby organs (eg, lung or gastrointestinal tract). In general, SBRT is very well tolerated and can even be performed on an outpatient basis.

Until more data about long-term safety and efficacy become available, it is prudent to limit this novel, palliative therapy to patients who have exhausted options of traditional, proven therapies for ventricular arrhythmias, including heart failure optimization, anti-arrhythmic medications, catheter ablation, and autonomic modulation. Furthermore, it is our practice at our institution to engage in an interdisciplinary discussion with the advanced heart failure team, as recalcitrant ventricular arrhythmias generally warrant consideration of heart transplantation or left ventricular assist device should SBRT fail to control the arrhythmia.

## Treatment planning and radiation delivery

The generally accepted dose for radiation therapy is 25 Gy delivered in a single fraction. The ability to target the correct arrhythmogenic substrate is of paramount importance and one that requires an interdisciplinary approach combining cardiac electrophysiology, radiology, and radiation oncology. A number of different methods have been reported to localize ventricular arrhythmias and correlate these to available cardiac imaging studies. Some centers utilize non-invasive electrocardiogram (ECG) imaging (ECGi) with a 256-electrode vest, which the patient wears during programmed stimulation via a previously placed cardiac implantable electronic device (CIED) in order to induce and map the arrhythmia.^2^ Other investigators have relied on invasive electroanatomic mapping data during catheter ablation procedures to identify the critical sites for targeting.^3^ Additionally, the integration of cardiac magnetic resonance imaging (MRI) data with late gadolinium enhancement for detailed scar delineation can also help guide the planning process.^5^ The readily available 17-segment model, often employed in cardiac imaging, can be utilized to streamline the targeting process and provide a common vocabulary between radiation experts and cardiac electrophysiologists.^15^

**Figure 1:**
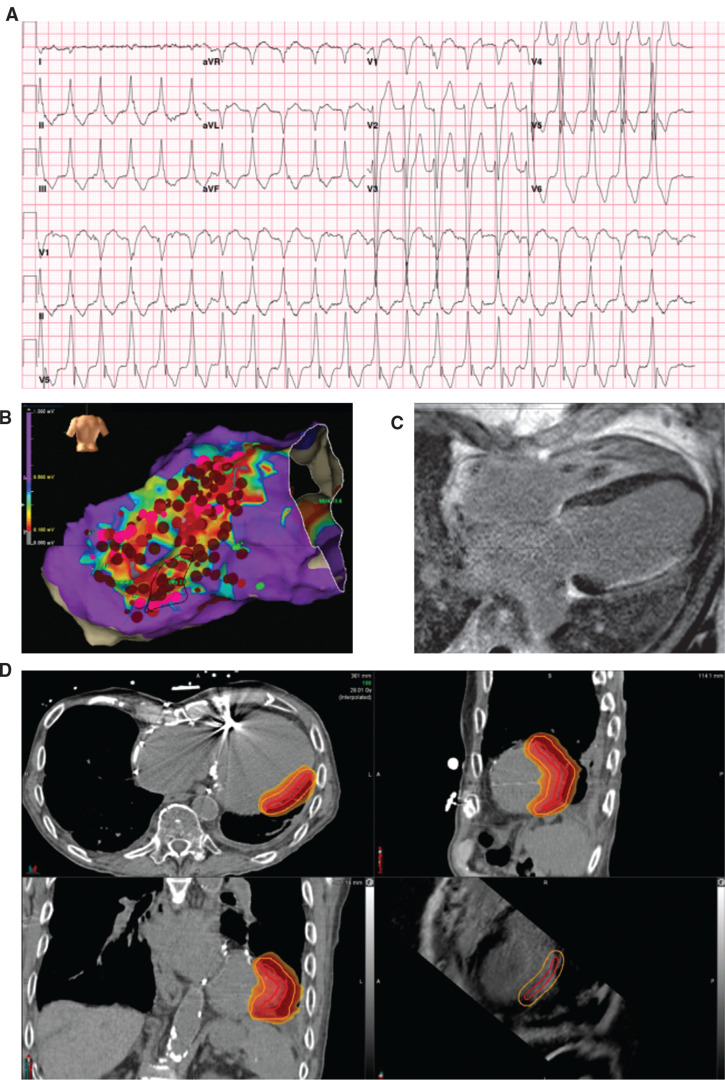
Stereotactic body radiation therapy planning case example. An 84-year-old man with a history of ischemic cardiomyopathy presented with recurrent monomorphic ventricular tachycardia (VT). **A:** A 12-lead ECG of VT morphology with left bundle branch block, right inferior axis, and V5 transition consistent with a mid-to-apical anterolateral exit. **B:** Posterior view of the electroanatomic voltage map from prior catheter ablation procedure (NavX™; Abbott, Chicago, IL, USA) at dense scar settings (0.1–0.5 mV). **C:** Contrast-enhanced cardiac magnetic resonance image with wide-band sequencing demonstrating late gadolinium enhancement of lateral left ventricular wall consistent with prior circumflex infarct. **D:** Radiation therapy planning computed tomography images with isodose contour lines indicating the targeted region.

The patient undergoes a computed tomography (CT) simulation scan with a 1.5-mm slice thickness as well as a 4-dimensional CT for respiratory motion assessment. These images are then combined with the reference data (ie, 12-lead ECG, echocardiography, ECGi, MRI, electroanatomic mapping), and the gross target volume is identified on the simulation images via discussion between radiation physicians and electrophysiologists. This is then used to generate the planning target volume (PTV), which is typically expanded slightly beyond the target region in order to account for motion and other uncertainties. The radiation treatment plan is optimized to deliver the prescribed radiation dose to PTV while minimizing exposure to organs at risk (OARs). **Figure 1** shows an example of a patient with PTV delineated on planning CT images after the incorporation of surface 12-lead ECG morphology of VT, electroanatomic mapping, and late gadolinium–enhanced cardiac MRI.

The first-in-man report of SBRT for ventricular arrhythmias utilized 25 Gy delivered in a single fraction.^16^ As multiple case series have followed, nearly all have followed suit with a similar regimen of 25-Gy dose selection.^2–6^ Limited animal model data have suggested this as the optimal dose; however, it is important to note that these studies were primarily investigating the effects on atrial tissue.^11,17^ Other animal studies have suggested that higher doses may in fact be required to achieve the desired electrophysiologic effect.^12,14,18,19^ Data from lung cancer studies have indicated that cardiac radiation doses of 40 Gy or more have been associated with reduced overall survival and should therefore be avoided.^20^ However, it is important to note that the radiation delivery strategy for lung cancer (radiation dose divided over multiple fractions) is much different than that employed for arrhythmia therapeutics (single fraction).

Various methods have been developed to ensure accurate delivery to the targeted tissue and are reviewed extensively elsewhere.^21^ Vacuum cushions, such as the BodyFix system reported by the original Electrophysiologic-guided Noninvasive Cardiac Radioablation for Treatment of Ventricular Tachycardia (ENCORE-VT) trial, are commonly used to immobilize the patient during radiation delivery.^2^ An onboard image guidance system, such as cone-beam CT, is necessary for accurate patient positioning before beam delivery. Abdominal compression can be used to reduce organ respiratory motion. The CyberKnife (Accuray, Inc., Sunnyvale, CA, USA) technology utilizes a fiducial marker such as a temporary pacing wire or the lead of a CIED with gating of radiation delivery to ensure accuracy.^3,4^ Patients with a CIED should have their devices interrogated and reprogrammed as appropriate before and after SBRT delivery, in accordance with task force recommendations.^22^

## Toxicities and long-term safety

Radiation-associated cardiac disease has been well described, owing to the off-target effects of cancer radiation therapy such as for breast cancer, lymphoma, or lung cancer. Radiation-associated cardiac disease can present with various manifestations.^23^ Restrictive cardiomyopathy, likely owing to myocardial fibrosis, can result in worsening heart failure with preserved ejection fraction, though systolic dysfunction can also occur. Valvular dysfunction due to worsening thickening and calcification of heart valves can cause worsening stenosis or regurgitation. Pericardial disease can result in both acute and chronic pericardial calcification, and thickening can lead to constrictive physiology. Vasculopathy may also occur, classically affecting the ostia or proximal coronary arteries. Finally, radiation can also affect the conduction system, causing anything from sinus node dysfunction to infra-Hisian conduction blocks. With the exception of acute pericarditis, many of these radiation-associated cardiac diseases develop years or decades after exposure. Thus, while faced with the more acute life-threatening consequences of ventricular arrhythmias, the risk–benefit profile may favor SBRT, though these are important considerations when applying this novel therapy to potentially younger, healthier patients. Adverse events of published clinical case series to date are included in **Table 1**.

OARs are an important consideration when targeting the cardiac tissue for SBRT. Adjacent organs vulnerable to the off-target effects of radiation therapy include the gastrointestinal organs (stomach, bowel, esophagus), lung, bronchi, spinal cord, ribs, and pericardium. Taskforce consensus recommendations should be followed to ensure that these OARs do not receive an excessive dose within the field of SBRT.^24^ The maximum point doses for these organs are included in **Table 2**. Pericarditis and pneumonitis are common early adverse effects, which have been reported in multiple case series. In the longer-term report of the ENCORE-VT trial, which is the largest series to date consisting of 19 patients, at a median follow-up of 23.5 months, 1 patient developed pericarditis within 90 days, 2 patients developed pericardial effusions >2 years after SBRT, and 1 patient developed a gastropericardial fistula 2.4 years after SBRT which required surgical correction.^25^ These toxicities, both cardiac and non-cardiac, will be important to keep in mind as more long-term data become available on patients receiving SBRT for cardiac arrhythmias.

## Unanswered questions

Published case series have reported variable degrees of success with varying time courses of efficacy. The presumed mechanism of action has been principally myocardial necrosis with subsequent fibrosis, thus eliminating critical VT circuits. However, multiple studies have reported early efficacy almost immediately after SBRT delivery, which contradicts this hypothesis. In the case series published by Gianni et al.,^4^ 3 patients underwent repeat catheter ablation procedures after SBRT. In all patients, there were low-voltage, fractionated potentials within the dense scar regions, which should have theoretically been eliminated with SBRT. If not by myocardial tissue destruction and fibrosis, then alternative hypotheses must be considered.

In a rat model, ionizing radiation was delivered in escalating doses and a histopathological analysis was performed within 4 weeks of exposure. While myocardial necrosis and apoptosis were not observed, disruption of intercalated discs with myocyte vacuolization and intracellular and extracellular edema occurred.^26^ Conduction slowing was also evidenced by surface ECG characteristics. This might serve as a potential explanation for the early benefits of SBRT reported in certain studies.^2,6^ These findings are supported by histopathologic findings of 3 patients after explantation during heart transplant showing minimal fibrosis but significant compromise of intercalated discs.^7^

Another recent animal study offers an explanation that conflicts with the scar-homogenization theory of SBRT. In a canine infarct model, delivery of 15 Gy via ^12^C radiation resulted in the reversal of conduction slowing and reduction in VT/ventricular fibrillation inducibility, with the upregulation of connexin 43.^27^ Left ventricular systolic function was also improved with radiation delivery compared to the untreated animals with myocardial infarction, which was thought to be due to increased connexin 43 expression within the infarcted regions.

## Conclusion

SBRT is an emerging therapy that shows great promise in the management of ventricular arrhythmias. While initial case series have demonstrated encouraging results, many questions remain as to the mechanism of action, time course of benefit, optimal method of delivery, dose selection, and long-term efficacy and safety. Thoughtful, rigorous randomized clinical trials and continued translational work will be critical to answer many of these important questions.

## Figures and Tables

**Table 1:** Published Case Series to Date of Stereotactic Body Radiation Therapy

Author	Publication Date	Number of Patients	Radiation Dose	Treatment Modality	Outcomes	Adverse SBRT-related Events
Cuculich et al.^2^	2017	5	25 Gy	C-arm linac	VT burden reduction in all, one non-arrhythmic death	None
Neuwirth et al.^3^	2019	10	25 Gy	CyberKnife	3/10 with electrical storm recurrence	Delayed: 1 case of worsened mitral regurgitation
Gianni et al.^4^	2020	5	25 Gy	CyberKnife	3/5 with VT recurrence requiring ablation	None
Chin et al.^5^	2020	8	15–25 Gy	C-arm linac	3/8 with clinical benefit	None
Robinson et al.^6^	2019	19	25 Gy	C-arm linac	VT or PVC burden reduction in 17/18 evaluable patients	Acute: 1 case of pericarditis Delayed: 2 cases of pericardial effusion and 1 case of gastropericardial fistula
Lloyd et al.^7^	2020	10	25 Gy	C-arm linac	5/10 underwent transplant or hospice care	2 cases of pneumonitis

*Abbreviations*: PVC, premature ventricular contraction; SBRT, stereotactic body radiation therapy; VT, ventricular tachycardia.

**Table 2:** Organs at Risk During Cardiac Stereotactic Body Radiation Therapy and Associated Dose Constraint and Maximum Point Dose Values According to Task Force Recommendations^24^

Organ	Volumetric Constraint	Maximum Point Dose (Gy)
Esophagus	V11.9 Gy < 5 mL	15.4
Spinal cord	V10 Gy < 0.35 mL	14
Rib	V22 Gy < 1 mL	30
Skin	V23 Gy < 10 mL	26
Stomach/duodenum	V11.2 Gy < 5 mL	12.4
Bowel	V11.9 Gy< 5 mL	15.4
Trachea/large bronchus	V10.5 Gy < 4 mL	20.2
Pericardium	V16 Gy < 15 mL	22
Lungs	More than 1,500 mL < 7 Gy	NA

*Abbreviations*: NA, not applicable; SBRT, stereotactic body radiation therapy.
